# 

**Published:** 1856-01

**Authors:** 


					﻿COMMUNICATIONS FOR PUBLICATION TO BE ADDRESSED TO ‘EDITORIAL COMMITTEE.’
IOWA
MEDICAL JOURNAL.
CONDUCTED BY THE FACULTY OF THE
COLLEGE OF PHYSICIANS AND SURGEONS
OF TtlE
IOWA. UNIVERSITY.
£
O
a!
E-
<1
Pi
; a I
i a
<
>
A
>—
a
e
a
Ed
o
o
o
t-1
f
>
w
CO
>
O ■
>
a
FOL. III. DECEMBER AND JANUARY, 1855-6. NO. 1.
EDITORIAL COMMITTER,
D. L. M’GUGIN, M. D .
JNO. R. ALLEN, M. D.
FINANCE committee.
J. C. HUGHES, M. D.
KEOKUK, IOWA.
PRINTED AT THE POST BOOK AND JOB OFFICE.
BUSINESS COMMUNICATIONS TO THE ‘FINANCE COMMITTEE.’
				

